# Enhancing maize growth and drought resilience by synergistic application of plant growth promoting rhizobacteria

**DOI:** 10.1038/s41598-025-29110-2

**Published:** 2025-11-27

**Authors:** Priyanka Khati, Pankaj Kumar Mishra, Ramesh Singh Pal, Lakshmi Kant

**Affiliations:** ICAR VPKAS, Almora, Uttarakhand 263601 India

**Keywords:** Drought stress, PGPR, Plant microbe interaction, *Zea Mays* L., Maize, Rainfed agriculture, Microbial bioinoculants, Microbiology, Plant sciences

## Abstract

The agriculture system in India is mostly rainfed, water is a major limiting factor in the country. Limited access to available water creates a drought-like condition which severely affects the crop production. Maize was selected as the target crop due to its importance as third major crop after wheat and rice and its huge water demand. The present study was conducted to observe the effect of bacterial inoculants on drought stress tolerance of *Zea mays* L. The maize seeds (DH line and composite) were treated with 2 bacterial inoculants (LZn-4 and S34) which were characterized as plant growth-promoting microorganisms and were able to tolerate water potential of − 1.5 MPa. The experiment was conducted in two conditions *Vis-à-vis* stress and without stress. As per the observation plant height, relative water content, antioxidants level, and soil enzymes were found to be maximum in the case of LZn-4 in drought conditions. Principal component analysis also highlighted the positive correlation of some of the treatments (T2 and T8) with microbial inoculants towards different stress related factors. The present investigation needs to be taken to the field level to understand the relationship between microbial inoculants and drought stress in real-time situations.

## Introduction

Agriculture in India is mainly rainfed which leads to the dependence of agriculture on the occurrence of rain. Climate change in recent years has severely affected the agriculture production system as now the occurrence of rain has become unpredictable causing a drought-like situation in the region. Drought stress is one of the most limiting factors for the growth and productivity of crops. According to Németh et al.^1^ drought stress induces stomatal closure, and therefore reduces photosynthesis. Plant Growth promoting Rhizobacteria (PGPR) are known to initiate “induced systemic resistance (ISR)” under various biotic and abiotic stress conditions^2^. These PGPRs colonize the roots and stimulate plant growth through indirect and direct mechanisms^3^. The direct mechanism induces the secretion of various growth regulators on the other hand facilitates the uptake of nutrients or causes genetic modifications under the indirect mechanism^4–6^. According to Bensalim et al.^7^ the exopolysaccharide-secreting bacteria are known to protect plants against drought stress as they help in the improvement of soil aggregation leading to root adherence and high relative water content in leaves. Moreover, PGPR can stimulate root system architecture—enhancing root length, branching, and surface area—thereby improving the plant’s capacity to access deep soil moisture^7^.

The stress in plants affects the level of reactive oxygen species (ROS) for which plants synthesize antioxidant enzymes such as peroxidase (POX), ascorbate peroxidase (APX), catalase (CAT), etc.^8^. Drought stress has a direct effect on stomatal closure and opening and hence also affects the chlorophyll content and thus photosynthesis^9^.

The impact of PGPR on drought stress mitigation have been studied by various researchers on *Arabidopsis thaliana* through the inoculation of *Paenibacillus polymyxa*^10^ wheat through *Azospirillum brasilense* Sp245^11^, on sunflower by *Pseudomonas putida* strain GAP-P45^7^. These findings highlight the species-specific and strain-dependent nature of PGPR-plant interactions, emphasizing the need for crop-specific evaluation. The mechanism involved in drought stress mitigations are improvement in roots structure and volume which help in better acquisition of water and Exo-polyssacahrides (EPS) secretion which again help in better soil structure and its water holding structure^7^. Maize is among the most prominent crops for human consumption, and, together with rice and wheat, is the staple food in India. Maize is highly susceptible to drought, especially during the vegetative and reproductive growth stages. As a C4 plant, maize has relatively high water-use efficiency, yet severe water stress can drastically reduce grain yield and quality. Additionally, the high-input nature of modern maize cultivation, involving intensive use of chemical fertilizers and herbicides, contributes to soil degradation, salinization, and water shortages, further aggravating stress conditions^12^. These hazardous implications along with climate change are severely affecting crop productivity which necessitates new insights towards improving drought tolerance in different crops. Given these challenges, integrating biological solutions like PGPR into maize cultivation offers a sustainable alternative to conventional practices. PGPR-based bioinoculants not only reduce the dependency on agrochemicals but also enhance soil health and crop resilience to environmental stresses. Therefore, considering all the highlighted points, the current research aimed to study the impact of PGPR isolates on the drought stress tolerance capability of *Zea mays* under artificially induced drought conditions. The study focuses on physiological, biochemical, and morphological parameters to evaluate the mechanisms by which PGPR confer drought resilience in maize.

## Results

### Impact of different treatments on plant health properties

The composite maize plants attained better height in comparison to DH lines. The plants under stress or without stress did not show any clear difference in terms of growth pattern among different treatments, which may be due to the glass house effect causing uncontrolled and uneven axial growth in maize plants. On the other hand, better leaf structure and number were observed in plants treated with bacterial inoculants under drought conditions. Relative water content is the measure of water level maintained in different parts of plants. As per the observation (*V1D1B1*), LZn4 treated composite seeds under drought conditions showed the best RWC (61.61) which was about 3 times higher than the control. Similarly in the case of normal conditions (*V1D2B1*) was observed to be maximal (30.98) which was 1.5 times greater than the control (Table 1). No clear pattern was observed in plant height and number of leaves concerning treatments applied.

**Table 1:** Plant physical characteristics.

	Relative water content (%)	Plant height (cm)	Number of leaves
V1D1B0	22.34 ± 3.82	182.5 ± 4.33	9.75 ± 0.48
V1D1B1	61.61 ± 13.45	170 ± 6.77	10.75 ± 0.75
V1D1B2	24.60 ± 6.26	199.5 ± 7.38	13.25 ± 1.44
V1D2B0	21.14 ± 3.61	230.25 ± 8.07	14.5 ± 0.50
V1D2B1	30.98 ± 1.47	218.75 ± 8.8	13 ± 0.71
V1D1B2	29.91 ± 5.23	216.25 ± 5.54	9.25 ± 0.58
V2D1B0	21.13 ± 4.11	121.25 ± 4.4	8.5 ± 1.71
V2D1B1	27.37 ± 9.04	180 ± 5.40	12.5 ± 0.29
V2D1B2	12.69 ± 2.69	115 ± 2.04	9.5 ± 0.50
V2D2B0	20.40 ± 3.06	190 ± 2.04	12.5 ± 0.29
V2D2B1	14.39 ± 3.09	151.25 ± 2.39	12 ± 0.71
V2D2B2	25.74 ± 6.90	177.5 ± 3.23	12 ± 0.71

### Impact of different treatments on roots structure

The impact of different treatment on root dynamics under drought and no stress was studied through root scanning.

**Fig. 1:**
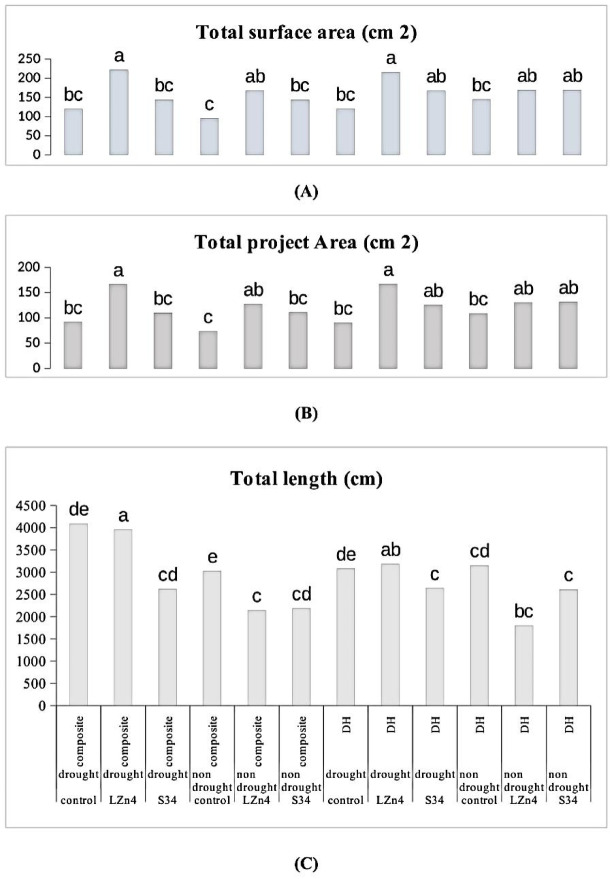
Bar diagrams to show root parameters as affected by drought stress and inoculation of bacterial inoculum.

It was observed that V1D1B1 (Composite + LZn4 + stress) has maximal total project area (166.08 cm^2^) and total surface area (221.95 cm^2^) and second highest total root length (3955.34 cm) which were at par with V2D1B1 (DH + LZn4 + stress) with total root length (3182.09 cm), total project area (166.89 cm^2^) and total surface area (215.67 cm^2^). Similar pattern was also obtained in case of normal conditions but the stressed plants when treated with bacterial inoculants (LZn4) performed best among all (Fig. 1).

### Impact of stress on antioxidant enzymes

The antioxidant enzymes are indicators of stress regulation, which are synthesized to scavenge the reactive oxygen species and free radicals.

**Fig. 2:**
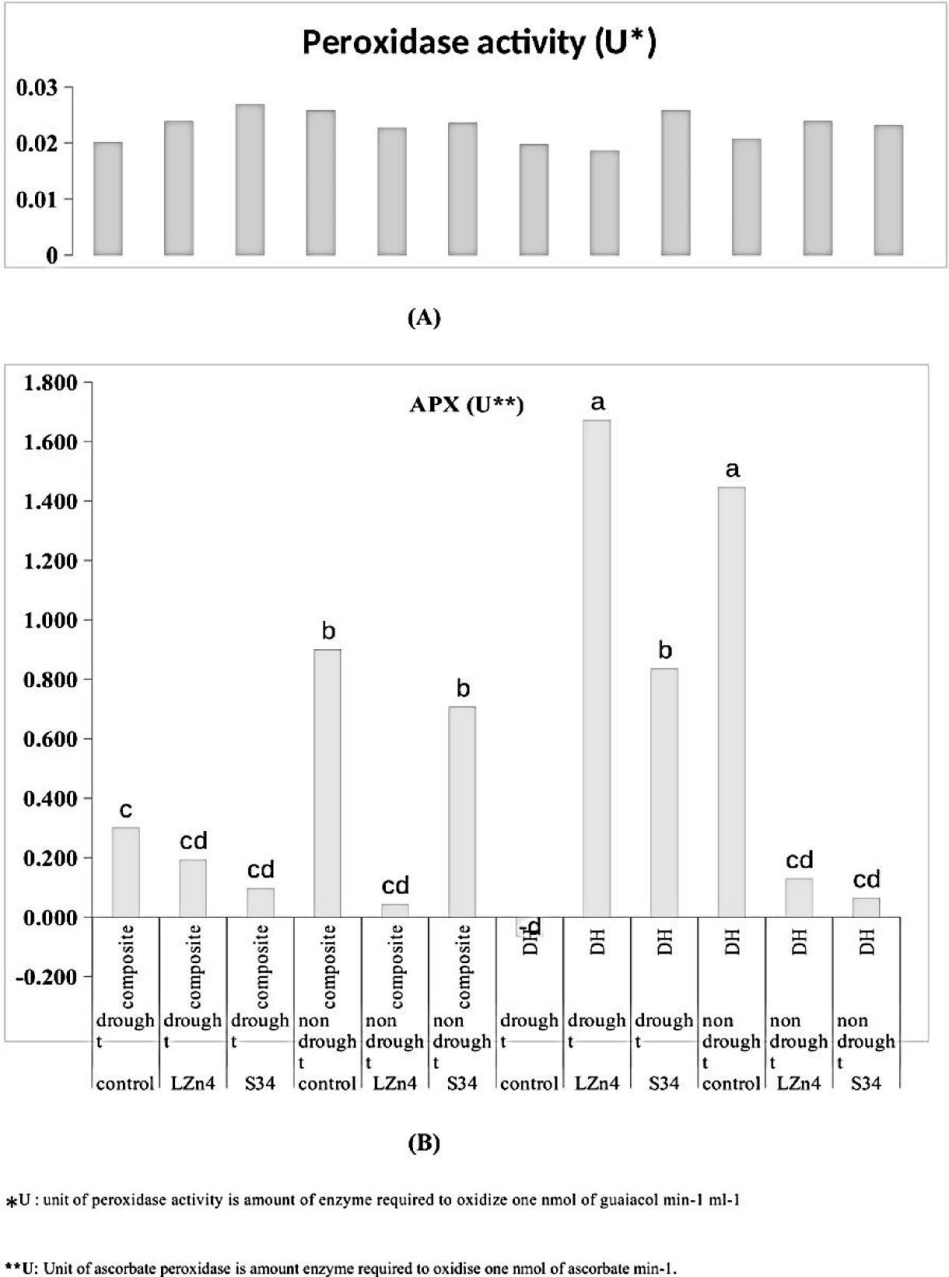
Impact of different treatments on plant stress tolerance through (**A**) peroxidase (non-significant) and (**B**) Ascorbate peroxidase activity (APX).

The activity of peroxidase was highest in *VIDIB2* (0.026 unit) followed by *V2D1B2* (0.025 unit) which were non-significant. The non-significant difference shows no response of peroxidase activity towards the treatments. On the other hand, Ascorbate peroxidase was found to be highest in the case of *V2D1B1* (1.671 unit) whereas it was least in the case of *V2D1B0* (− 0.06 unit) (Fig. 2). The results show the microbial inoculum in case of drought stress has caused a hike in antioxidant enzymes to improve the stress tolerance mechanisms under the situation.

### Soil physicochemical and biochemical properties

#### Physicochemical properties

The soil was slightly acidic which did not change even after the treatments. No difference in soil moisture content was observed in normal pots with no stress before and after the treatments but in case of stressed pots some reduction in soil moisture content from The electrical conductivity of the soil lies in low range (80 µS) in the initial sample. The EC after the experimentation was still in low range but higher than the initial reading and varied slightly with the different treatments. It was highest in *V1D1B1* (210 µS) and followed by *V2D1B1* (207 µS) whereas the least was observed in *V1D1B0* (88.1 µS). The electrical conductivity in non-stressed plants was poor in comparison to stressed plants (Table 2). The electric conductivity is a measure of dissolved solutes which normally lies below 4000 µS. The results show there was no negative impact of drought on soil.

**Table 2:** Soil physicochemical and biochemical properties.

	Composite						DH line					
	V1D1B0	V1D1B1	V1D1B2	V1D2B0	V1D2B1	V1D2B2	V2D1B0	V2D1B1	V2D1B2	V2D2B0	V2D2B1	V2D2B2
pH	Slightly acidic											
EC (µS)	88.1	210	117.1	98.5	124	101.8	173.3	207	131.2	111	147	98
Total bacteria (10^6^)	5.8	6.1	5.3	10.7	7.2	7.1	5.9	6.5	7.2	8.0	4.7	3.1
Psuedomonads (10^6^)	3.6	1.6	4.9	7.5	5.2	6.0	4.6	3.0	3.4	6.4	4.5	2.8
Actinomycetes (10^6^)	1.67	2.07	1.56	2.9	1.48	1.55	1.78	1.46	1.45	2.09	1.86	1.25
Azotobacter (10^6^)	1.47	2.40	2.99	2.99	0.62	2.84	0.36	2.96	0.47	2.98	1.78	2.05

#### Soil microbial population dynamics

The bacterial (107 × 10^5^) and Pseudomonad population (75 × 10^5^) was found to be highest in case of non-stress conditions as they can be directly affected by drought stress on the other hand *Actinomycetes* (300 × 10^4^) and *Azotobacter* (300 × 10^4^) population which are known for their stress tolerance capabilities were comparable in both the situation of stress in composite. In case of in DH line best microbial population was shown by LZn4 inoculated treatments in drought stress condition (Table 2).

#### Soil enzymes

**Fig. 3:**
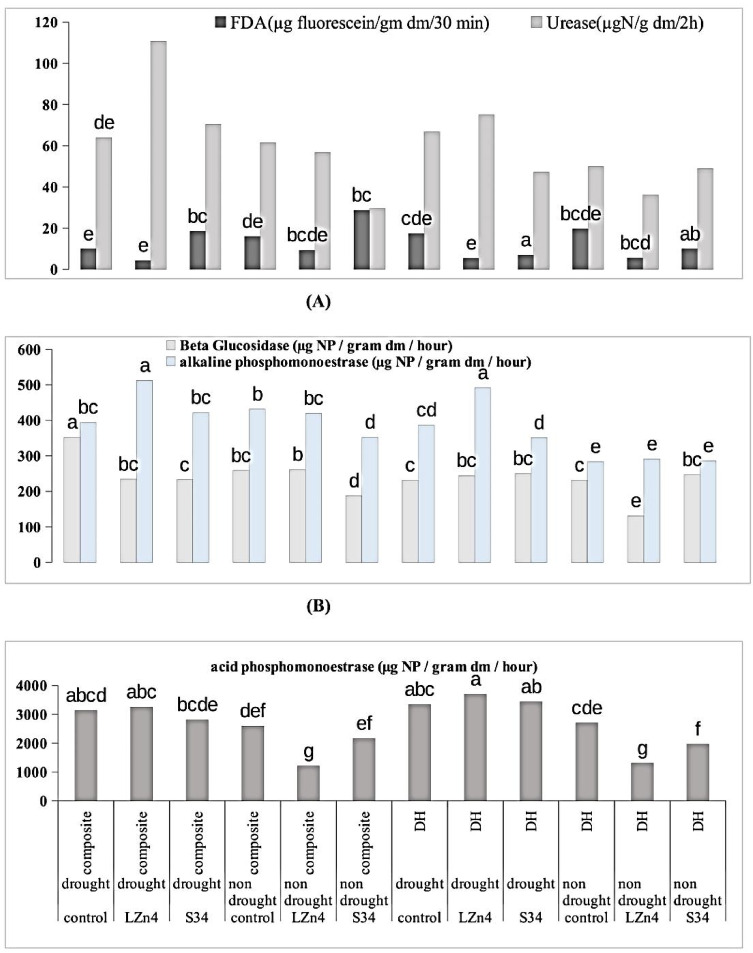
Enzyme activities (**A**) for Fluorescein diacetate (FDA) and Urease and (**B**) alkaline phosphomonoesterase and B-glucosidase (**C**) Acid phosphomonoesterase.

The highest urease activity was recorded in *V1D1B1*(110.85 U) followed by *V2D1B1* (75.05 U) whereas *V1D1B0* and *V2D1B0* were observed to be 63.94 U and 66.66 U respectively. The other control treatments without stress showed better urease activity i.e. 61.49 U and 49.87 U for *V1D2B0* and *V2D2B0* respectively except *V1D2B1* (56.78 U) which was able to maintain the activity due to bacterial inoculum (LZn4) (Fig. 3A).

In the case of FDA activity, in composite maize high activity was recorded in S34-treated plants in stress (18.52 U) or without stress (28.74 U) in comparison to LZn4 treated or the control plants. Whereas in the case of DH line control received maximum FDA activity with 17.4 U in a stressed condition and 19.7 U in non-stressed condition respectively. Highest β-glucosidase activity was observed in V1D1B0 (352.6 U) and the range varied from 352 to 131 U approximately (Fig. 3B).

Alkaline phosphomonoesterase activity was highest in case of *V1D1B1* (512.2 U) and acid phosphoestrase activity was highest in *V2D1B1* (3698.06 U) in stressed condition (Fig. 3B, C). Whereas in case of without stress condition the enzyme activity was lower in both the varieties. Impact of drought stress on silk and tassel emergence.

The impact of stress on delaying in silk and tassel emergence was evident as in the case of DH lines silk and tassel emergence was hampered in drought stress. In the case of composite plants maximum time for tassel, (80 DAS) and silk (82 DAS) emergence was observed in control (*V1D2B0*) without any induced stress, which was a delayed emergence due to glasshouse conditions. Similarly for control in drought stress (*V1D1B0*) the tassel and silk emergence was normal and other treatments under drought for composite were at par.

**Fig. 4:**
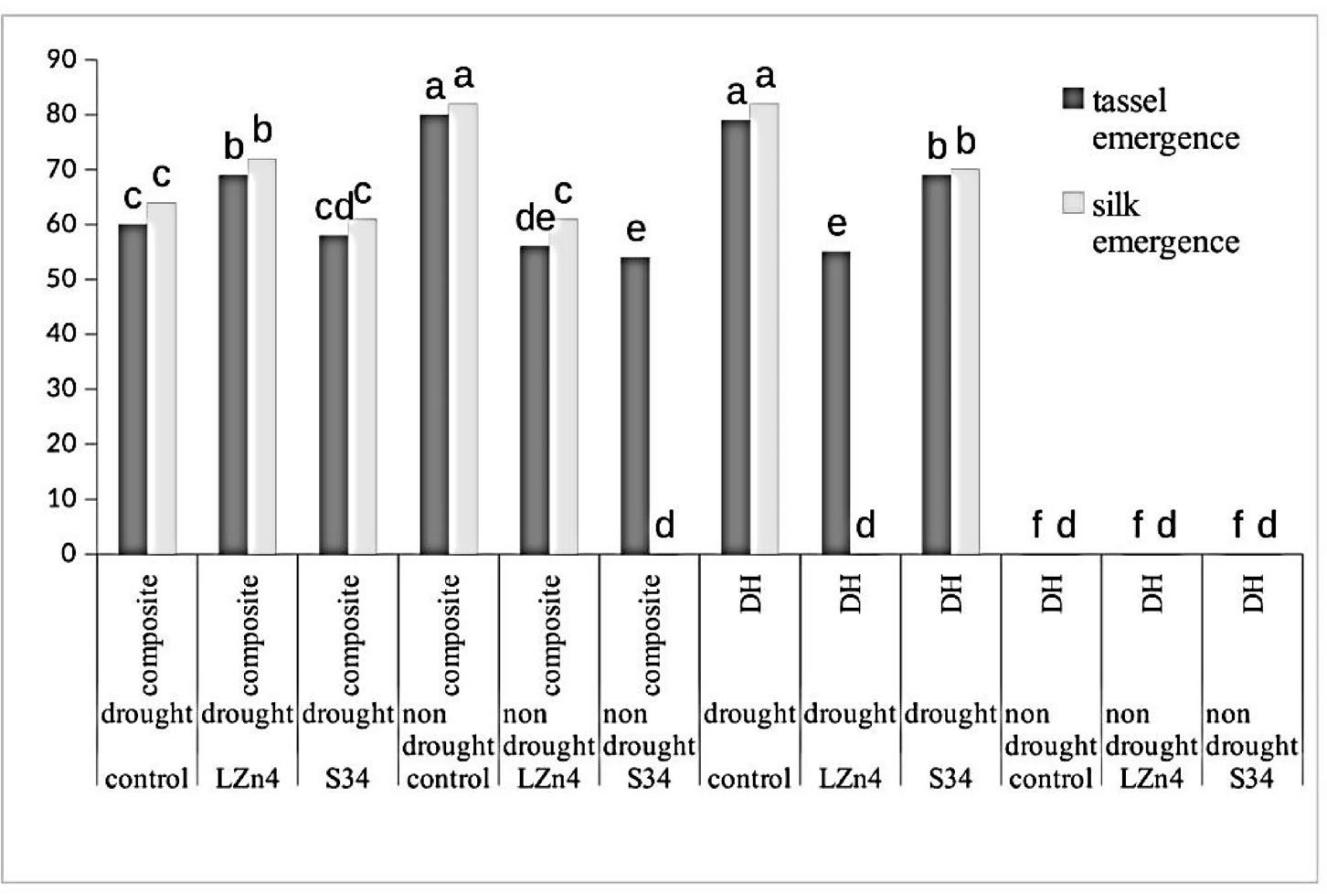
Bar diagram to show tassel and silk emergence duration in different treatments.

It can also be observed that reproductive growth in normal conditions was almost similar to stressed conditions in composite plants (Fig. 4).

### Principal component analysis

**Fig. 5:**
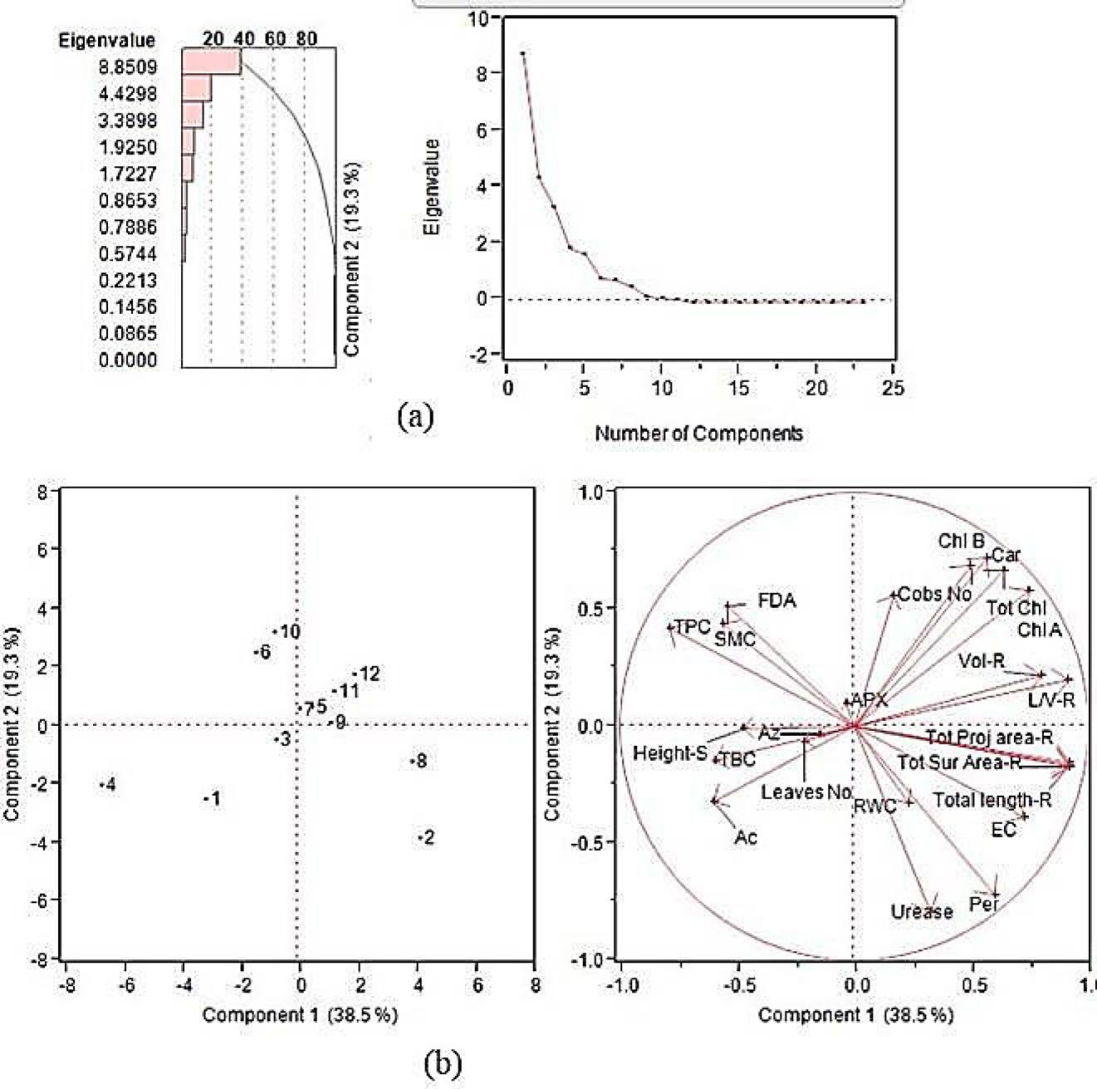
(**a**) Eigen value to represent the variability in the data and (**b**) Biplot analysis to show the relationships among the treatments (T1 to T12) and contribution of different variable towards Principal component. The different variables are: SMC, Soil microbial count; TPC, Total pseudomonas count; Az, Azotobacter count; TBC, Total bacterial count; Ac, Actinomycetes count in rhizospheric soil; FDA, Fluorescein diacetate; Urease content in soil; RWC, Relative water content in leaves; APX, Ascorbate peroxidase; Per, Peroxidase; Chl A, Chlorophyll A; Chl B, Chlorophyll B; Tot Chl, Total chlorophyll; Car, Carotenoid content in leave; EC, Electric conductivity in leaves samples; Height S, Height shoot; Leaves No, Number of total Leaves; total length-R, Total root length; total Sur Area-R, Total root surface area; total Proj area-R, Total root project area; L/V-R, Length per volume ratio of roots; Vol-R, Root volume; Cobs No, Number of cob per plant.

The PCA analysis shows the negative impact of treatments T1, T3, and T4 on the number of leaves, shoot length, counts of *Azotobacter*, *Actinomycetes*, and total bacteria. Treatments T2 and T8 had positive impact on Relative Water Content, Electric Conductivity, urease activity, peroxidase activity, total length, total surface area, and total project area of roots. Treatment T7 lies in the border which is a control with drought stress in DH line. Treatment T5, T9, T11 and T12 responded positively towards volume and length per volume of roots. The same treatments also showed a positive response towards number of cobs, chlorophyll and carotenoid content in plants. On the other hand treatments T6 and T10 had a negative interaction with soil microbial count (SMC), total pseudomonas count (TPC), Fluorescein diacetate (FDA), and Ascorbate peroxidase (APX) unit (Fig. 5).

**Fig. 6:**
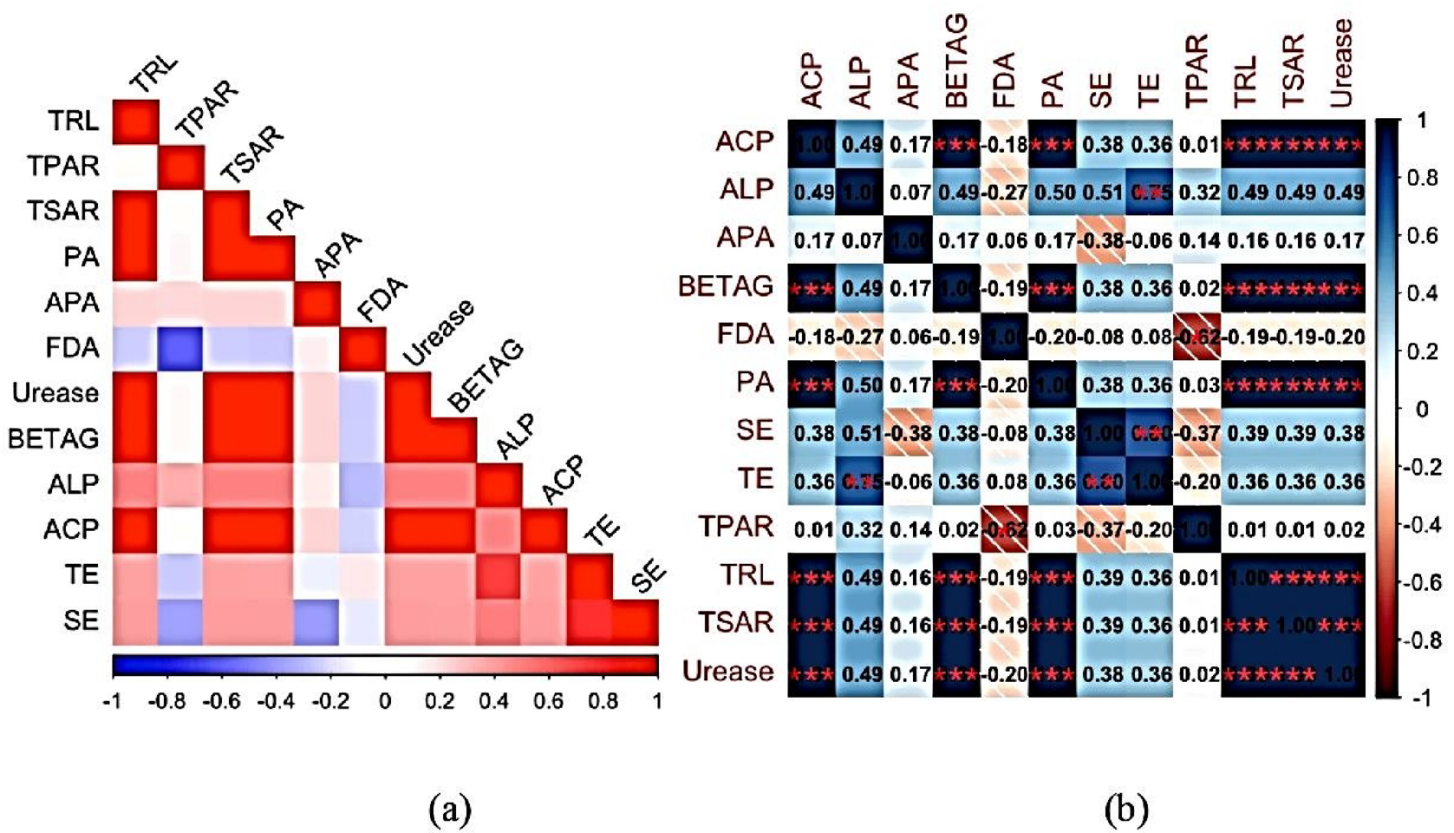
(**a**) Correlation plot, (**b**) Correlation matrix showing pairwise Pearson coefficients between different parameters studies. TRL, Total root length; TPAR, total project area root; TSAR, Total surface area root; PA, Peroxidase activity; APA, Ascorbate peroxidase activity; FDA, Fluorescein diacetate activity; Urease, Urease activity; BETAG, Beta Glucosidase activity; ALP, Alkaline phosphatase activity; ACP, Acid phosphatase activity; TE, Tassel emergence; SE, silk emergence. *Level of significance was set at 0.001, 0.01 and d 0.05.

Pearson’s correlation analysis was conducted to investigate the relationship between growth parameters, biochemicals, and antioxidant activity in normal and stress conditions. The silk and tassel emergence (SE and TE) were showing positive correlation with TRL (total root length), TSAR (total surface are), PA (peroxidase activity of soil), and different soil enzyme activities such as Beta glucosidase (BETAG), alkaline phosphatase (ALP), acid phosphatase (ACP) (Fig. 6a). The results show significance in ACP with BETAG, PA, TRL, TSAR and Urease at all there level (α = 0.001, 0.01 and 0.05). ALP was significantly related to TE at 0.01 and 0.05 of significance level. BETAG was significantly related to ACP, PA, TRL, TSAR and Urease at all there levels (Fig. 6b). The results shows maximum correlation among the root parameters (TPAR, TRL and TSAR) and soil enzymes (Urease, acid phosphatase, and BETAG) suggesting there significance in drought stress response in maize.

## Discussion

The plant height and leaves number were irresponsible to different microbial treatments which may be due to the effect of glass house causing intense radiation and higher axial growth in some treatments. Relative water content was highest in composite plants treated with LZn-4 under both conditions which highlights the water conservation nature of plants treated with bacterial inoculum. The relative water content shows the balance between the water supply and the rate of transpiration in leaves^13^. The plants under drought stress showed more relative water content than the plants under normal conditions. The results show that drought condition leads to more water conservation in plants treated with bacterial inoculants. Similar kind of results were obtained by Batool et al.^14^ as they observed an improvement in relative water content in the case of plants treated with PGPR.

The bacterial strains LZn4 and S34 as per our previous study^15^ were observed to have good phosphorous (P) and zinc (Zn) solubilization potential along with siderophore production. The PGPRs improve plant health through different mechanisms (Zn solubilization, P solubilization and siderophore production etc.) which indirectly help the plant to tolerate stress like conditions. The improved plant health and vigour make them tolerant to different kind of stresses. The microbes interact with plants and provide a hemostasis for ion exchange and thus maintaining nutritional status of plant under drought stress^16^.

On the other hand microbes also releases several organic and inorganic components which are used as signalling molecules and nutritional component under stressed conditions^17,18^. The bacterial isolates with P solubilization capabilities can also increase chlorophyll content, leading to improved photosynthesis. Higher P level also leads to higher proline and glycine betain content which help in regulation of drought stress^19^. Similarly in a study by Bilal et al.^20^ the impact of microbial isolates were investigated on Glycine max and positive improvement in dry biomass, root, shoot length and leaf area was observed along with increased calcium and phosphorous levels. The results show the positive implications of microbe mediated nutrient management in stress tolerance of plants.

The primary and secondary roots are important for the plant to acquire water, especially in drought conditions. The root system is a direct indicator of stress management. Total length, project area, and surface areas of roots were estimated to be highest in composite plants treated with LZn-4 during drought stress followed by S34 treatment in the same condition. When the treatments under different conditions were compared it was observed that composite plants treated with LZn4 showed the best root development in both the conditions (i.e. drought and normal) followed by S34 treatment. Composite plants were longer in height and had a more severe rooting system in comparison to DH line. Overall results indicate improvement in root structure after bacterial inoculations as a mechanism of drought stress tolerance. The improved nutrition by microbial inoculation and better phytohormone regulations are also responsible for better root development and hence help in stress mitigation^21^. The results can be correlated to the previous results obtained by Abbasi et al.^22^ as they also linked the improvement in root and shoot biomass to bacterial inoculation during drought conditions in wheat and tomato respectively. Plants during stress conditions secrete several antioxidant enzymes to scavenge the reactive oxygen species (ROX) and free radicals. These antioxidant enzymes are fighting back mechanisms for tolerance and performance under stress conditions. Peroxidase activity was found to be highest in the case of composite plants treated with LZn4 under stress conditions followed by S34 treatment where other conditions were the same and the least activity was observed DH plant with no treatment and no stress. The indicators show an accumulation of antioxidant enzymes as a combat system under stressed conditions. Similarly carotenoid content was also high in treated plants. Similar results were obtained by Batool et al.^14^ observing improved antioxidant activity in stressed plants when treated with bacterial inoculum. In the case of APX maximum activity was observed in plant treated with S34 under drought stress although no clear relationship among the treated plants and stress condition was obtained.

The serial dilution method is the conventional yet most reliable method to deduce the total viable microbial population. The total bacterial population in case of drought stress was better when treated with bacterial strains, showing the positive response of the bacterial population towards treatments. Similarly, population of *Actinomycetes* and *Azotobacter* was observed best in inoculated treatments in both conditions (drought and without drought). The *Actinomycetes* are most resistant bacterial population in soil which justifies the impact of different treatment and drought stress on them. Similarly *Azotobacter* is a cyst forming bacteria which might help them to surpass the stress conditions causing their good growth and performance under stress. On the other hand pseudomonads population could not give a clear pattern among different treatments. According to Siebielec et al.^23^ drought had severe impact on population of microbial community and their activity. The sustenance of bacterial population even under extreme stress condition shows the potential of microbial inoculants to mitigate the drought stress conditions in soil. Secretion of Exopolyssacharides could be a possible mechanism behind, which improves the structure of soil and population of soil microorganisms through maintenance of moisture and nutrients^24^. Similarly Vurukonda et al.^25^ also studied the impact of PGP on drought stress tolerance of plants and found secretion of exopolyssacharides as one of the underlying mechanism.

Soil moisture content was less in stressed pots in comparison to non-stressed pots which was not even affected by any treatment. The electrical conductivity is the measure of total soluble solutes in soil and is good indicator of available nutrients. Soil electrical conductivity was better in pots under bacterial treatments which shows bacterial inoculants not only improve the plant stress responsive mechanisms but also improve and maintain the soil health through different mechanisms.

Enzymes are vital activators in the life cycle and they are known to play a substantial role in maintaining soil health. The soil enzymes play major biochemical functions in the process of organic matter decomposition^26^. The results of enzyme activities are also in correlation to other soil health-related parameters. Most of the enzymes showed the best results in the case of LZn4 treated samples in the case of both DH and composite seeds except in the case of β glucosidase which is a broad-spectrum enzyme and may also depend on other factors. According to Kiss et al ^27^. various substrates act as nutrients for microorganisms through decomposition. The phosphatase enhances P solubilisation when required^28,29^. Good enzymatic activity in soil is referred to as good microbial diversity and population. Vinhal-Freitas et al.^30^ also related an increase in enzyme activity to an increase in microbial biomass which again suggests that the soil enzymes are indirect indicators of soil microorganisms, and hence nano-chitosan application supports the microbial activity and soil health. The tassel and silk emergence showed a great impact of drought especially in the case of DH line but in composite the impact of drought was nullified with the help of microbial inoculants. Similarly, Khati et al.^31–33^ also studied the impact of nano-compounds on soil and plant health parameters of *Zea mays* L and found a positive correlation among different nano-compounds useful for soil and plant health with soil enzymes, microbial population, and other plant health parameters.

Agunbiade et al.^34^ isolated rhizobacteria strains from maize (*Zea mays* L.) and studied drought tolerance using polyethylene glycol (PEG)-8000 and other plant-growth-promoting activities. They characterized 11 bacterial isolates include *Bacillus licheniformis* A5-1, *Aeromonas caviae* A1-2, *A. veronii* C7_8, *B. cereus* B8-3, *P. endophytica* A10-11, *B. halotolerans* A9-10, *B. licheniformis* B9-5, *B. simplex* B15-6, *Priestia flexa* B12-4, *Priestia flexa* C6-7, and *Priestia aryabhattai* C1-9. Post inoculation of microbial inoculants increase in the above- and below-ground biomass of the maize plants at 100, 50, and 25% water-holding capacity (WHC) was observed. Similary in an another study by Azeem et al.^35^ impact of different *Bacillus* spp. Strains was studied on drought stress amelioration of Maize plants. Such bacteria could be used for enhancing the crop productivity and plant protection under biotic and abiotic stresses for sustainable agriculture. Co-inoculation of Pseudomonas sp. MRBP4 and Bacillus sp. MRBP10 on different maize genotypes also ameliorated the effect of drought stress. The increase in relative water content of the leaves of inoculated plants, and the soil moisture content was observed as a result of co-inoculation^36^.

The overall results show the importance of microbial inoculants especially LZn-4 in most case for improvement in soil enzyme activities, soil microbial population, and regulation of other physicochemical properties. The positive impact on plant root structure was observed in terms of total root length, surface area and project area which also lead to improved water uptake and stress resilience. In conclusion microbial inoculants in every aspect were observed to maintain plant and soil health during stress conditions. These microbial inoculants can be applied as formulations to address the water scarcity or drought stress in rainfed areas. Further studies at the field level need to be carried out to study the impact in real situations.

## Methods

### Maize seeds

Under present investigation, two genotypes (DH and composite) were selected as they both were drought susceptible. The selected two genotypes represent two different genetic background DKC7074DH-13 is completely homozygous and homogenous, whereas composite (C18) is heterozygous and heterogeneous. Maize Double haploid (DH) line (**DCK 7074, DH-13**) was received from Indian Council of Agricultural Research—Vivekananda Parvatiya Krishi Anusandhan Sansthan (ICAR VPKAS), Almora, Uttarakhand, which was well evaluated at Experimental farm, Hawalbagh, Almora, Uttarakhand (29°36′N, 79°40′E ) as well as winter Nursery Centre, Hyderabad) through phenotypic evaluation across multiple seasons/sites/statistical validation/comparative analysis with known standards, ensuring robustness and reproducibility of the results. On the other hand Composite (**C 8**) was obtained from Jammu and Kashmir, and observed to have drought susceptible nature under a research work done by Rashid as per his M Sc. thesis program^37^. These genotypes were identified as drought susceptible on the basis of phenotypic markers like tassel blast, leaf rolling and leaf firing at their respective testing centers.

### Bacterial inoculants

**Fig. 7:**
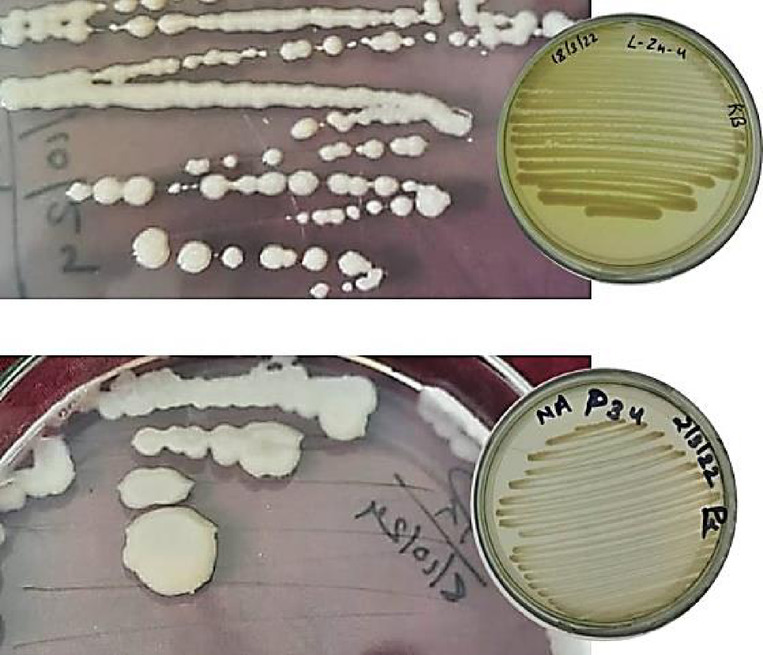
Plate morphology of bacterial strains LZn4 and S34.

The bacterial inoculants LZn-4 and S34 were isolated from lentil and Soybean rhizosphere at Hawalbagh (29° 38′ 37N latitude; 79° 38′ 7E longitude; 4189 feet Altitude) respectively were good PGPR (Zinc and P solubilization and siderophore production) with 35% of PEG-6000 tolerance^15^. These two strains were selected for the pot experimentation owing to their good PGP traits and tolerance towards water stress as tested on PEG-6000 amended medium. The DNA isolation was done with the help of isolation Kit (Cat No. MB505-50PR), which was amplified for 16srDNA region using universal bacterial primer (9bfm/1512uR) (initial denaturation at 96 °C for 4 min followed by 30 cycles of Denaturation (96 °C for 1 min), Annealing (52 °C for 1 min) and Extension (74 °C for 1 min) and a final extension at 74 °C for 10 min. The amplicon was sequenced through outsourcing with the help of Himedia (Cat No. MBS104). The read were subjected to BLAST and more than 98% similarity with the database was considered for identity of the isolates. The sequence was submitted to National Center for Biotechnology Information (NCBI). The LZn-4 and S34 were characterized as *Bacillus* sp. (Accession Number: OR880263) and *Acinetobacter* sp. (Accession Number: OR880260) respectively (Fig. 7)^15^.

### Pot trail

The experiment was designed with completely randomized design (CRD) (Factorial) with three replications. Following factors applied;

**Factor A** for seed Type **V1**: Composite (C8); **V2**: DH-DKC 7074 DH-13.

**Factor B** for Conditions **D1**: Drought stress; **D2**: No Drought.

**Factor C** for Bacterial inoculants: **B0**: None, **B1**: LZn4, **B2**: S34.

Degree of freedom was more than 12. Seeds of maize were washed thoroughly with tap water. Healthy seeds were surface sterilization for 2 min in 0.1% mercuric chloride solution and rinsed three times with sterilized distilled water to remove the residual traces of HgCl_2_. To prepare bacterial inoculum, single isolated colony was picked and inoculated in nutrient broth, which was incubated at 32 °C for overnight at 120 rpm shaking speed. The optical density (OD) of the inoculated nutrient medium was maintained at 1 at 600 nm in spectrophotometer. 0.1% carboxymethyl cellulose was added into the broth to enhance stickiness of broth. Ten ml of broth was added into 500 g of seeds and mixed thoroughly so that each seeds receive a uniform coating of bacterial population. Control seeds were not treated with bacterial inoculum which acted as positive control in case of drought stressed situation, whereas the treatment with inoculum (LZn4 and S34) without any stress acted as negative control (Table 1). Coated seeds were shade dried and four seeds per pot were sown.

The pot trial was conducted in unsterile soil under controlled green-house conditions, to precisely control environmental variables, particularly soil moisture. To maintain uniform growth condition the temperature was maintained at 28 ± 2 °C. The corn seeds were sown on 2 July 2022. One pot was sown with 4 seeds initially but they were thinned to one after 10 day after sowing (DAS) to maintain proper nutrient requirements in pots. Drought treatment was started after 15 DAS. To maintain drought conditions the pots were irrigated with 40% of its field capacity. The field capacity was calculated as per the standard method followed by Junker et al.^38^. The normal pots without induced stress were irrigated regularly with 100% field capacity. Fully matured crop was harvested and standard methods were adopted to collect the data. The pot house was maintained at 22–28 °C of temperature and 30–60% humidity during the entire experiment.

### Plant height, number of leaves, tassel and silk emergence

Labeled meter rod was used to measure plant height. Plant roots were taken up and washed with water and sodium Hexametaphosphate (10 g/L) to remove soil and scanned through root scanner to analyse the total root biomass.

### Plant physio-chemical properties

#### Relative water content

To measure relative water contents fresh weight of 0.5 g leaf was measured and soaked in test tube filled with water. Leaf portion was taken out from test tube after 24 h. Water droplets present on surface of leaf were removed by using tissue paper and leaf weight was calculated. The leaf sample was then dried up in an oven for 24 h at 80 °C and dry weigh was calculated. The relative water contents (RWC) were calculated as per the formula by Karrou and Maranville^39^

$${\mathrm{i}}.{\mathrm{e}}.\;{\mathrm{RWC}} = \left( {{\mathrm{FW}} - {\mathrm{DW}}} \right)/\left( {{\mathrm{TW}} - {\mathrm{DW}}} \right) \times {1}00$$

where FW is the fresh weight of sample, DW is the dry weight of sample and TW is the turgid/soaked weight of sample.

#### Plant antioxidant activity

The enzymes were extracted by macerating 25 g tissue with 90 ml of ice cold extraction medium in a pre-chilled pestle and mortar using acid washed sand as abrasive. The homogenate was filtered through four layers of cheese cloth and the filtrate centrifuged at 15,000 rpm for 20 min in a refrigerated centrifuge at 4ºC. The supernatant was carefully decanted and used as crude enzyme preparation. The extraction medium for POX was 0.1 M Tris–HCl buffer (pH 7.5) containing 3% (*w/v*) polyvinylpyrrolidone, 1 mM EDTA and 1 mM CaCl_2,_ whereas for APX 0.1 M potassium phosphate buffer (pH 7.5) in place of Tris–HCl buffer was used, the rest extract ants being the same.

*Peroxidase activity* 3 ml reaction mixture containing 0.1 ml of the enzyme extract, 0.4 ml of pyragallol in phosphate buffer and 0.5 ml of H_2_O_2_ were added in cuvette and change in absorbance was recorded at 420 nm for a period of 3 min. Control does not contain any enzyme extract. Enzyme activity was calculated by using 26.6 mM^−1^ cm^−1^ extinction coefficients^40^. One unit (U) of peroxidase activity was defined as the amount of enzyme required to oxidize one nmol of guaiacol min^−1^ ml^−1^.

*Ascorbate peroxidase (APX)* 3 ml of reaction mixture containing 1.42 ml (95 mM) potassium phosphate buffer (pH 7), 1.5 ml (0.5 mM) L-ascorbate, 50 µl (0.5 mM) H_2_O_2_ and 50 µl enzyme extract were added in cuvette and the change in absorbance was recorded at 290 nm for a period of 2 min. Control does not contain any enzyme extract. Enzyme activity was calculated by using 2.8 mM^−1^ cm^−1^extinction coefficients^41^. One enzyme unit was expressed as amount of enzyme required to oxidise one nmol of ascorbate min^−1^.

### Soil physicochemical properties

The soil is classified as silty clay loam. The soil pH was measured using pH meter after mixing soil: water ration (1:2.5) comprising 10 g of air dried soil^42^. Electrical conductivity was also measures using the same solution with the help of conductivity meter.

### Soil biochemical properties

#### Enumeration of total bacterial population

Enumeration was done by using standard method as described by Messer and Johnson^43^. (2000). Soil samples were diluted upto 10^4^ dilutions and pour plating was done using nutrient agar. Inoculated plates were incubated at 30° ± 1 °C in a BOD incubator for 24 h. Bacterial colonies were counted and expressed as log CFU g^−1^ of soil. This experiment was performed in triplicate.

#### Soil enzyme

*Fluorescein diacetate (FDA) activity* This activity indicates the hydrolysis of FDA by esterase, protease and lipase enzymes to release fluorescein which is a fluorescent indicator and gives indication for relevant population in soil. For this activity 1 g soil sample was placed in 150 ml flask with 50 ml sodium phosphate buffer (pH 7.6). Flasks were incubated after adding FDA solution (0.5 ml), in an orbital shaker for 1 h at 24 °C. Two ml acetone was added to terminate the reaction. Soil suspension was centrifuged at 8000 rpm for 5 min. Obtained supernatant was filtered using Whatman No.2 filter paper and absorbance was measured at 490 nm. FDA hydrolysis was expressed as μg flurorescein/g dry soil/h^44^.

*Alkaline and acid phosphatase activity* One gram of each soil sample was mixed with 0.25 ml toluene; 1 ml p-nitrophenyl phosphate (pNPP) (25 mM) and 4 ml of Modified universal buffer (MUB) (100 mM, pH 11) were added. After incubation at 37 °C for 1 h, one ml of CaCl_2_ (0.5 M) and 4 ml of tris buffer (0.1 M, pH 12) were added to stop the reaction. Intensity of the colour was determined using spectrophotometer at 400 nm. Para-nitrophenyl (pNP) was used as standard. Same procedure was also followed for acid phosphatase using the MUB working buffer with pH-6.5^45^.

*β-glucosidase activity* To 1 g dry soil, 0.25 ml of toluene, 1 ml of p-nitrophenyl-β-D-glucoside (pNPBG) and modified universal buffer (4 ml, pH 6.0) were added in test tube. Tubes were incubated at for 1 h at 37˚C. CaCl_2_ (1 ml, 0.5 M) and Tris buffer (4 ml with pH-12) were added in test tube. The suspension was left to develop colour and filtered before recording the absorbance at 410 nm. Enzyme activity was expressed as μg pNP released g^−1^ dry soil h^−1^^46^.

*Urease activity* 2.5 g of soil sample was taken in 150 ml flask containing 1.5 ml of substrate (Urea) and distilled water in control. The samples are incubated for 2 h at 370. After incubation 1.5 ml substrate was added in control and 1.5 ml of DW in treatment. 25 ml of KCL solution was added in every flask which is incubated for 30 min at 120 rpm shaking speed. The samples are filtered and 1 ml of filtrate was added into 2 ml of distilled water and 5 ml of Reagent A (sodium salicylate solution). Two ml of dichloroisocyanourate solution was evenly added into each tube. The tubes are incubated for next 30 min and reading was recorded at 660 nm^47^.

### Statistical analysis

The data in 3 replication was statistically analyzed through online software operational statistics (WASP 2) using completely randomized design. The *P* value was set < 0.05. The Principal component analysis (PCA) analysis was done to study the trend and correlation of different treatments with different parameters selected. In order to study the correlation among the different parameters, the correlation analysis was done at three different significance level (α = 0.001, 0.01 and 0.05) with the help of R software.

## Data Availability

The bacterial isolates are well characterized and submitted in NCBI with accession code OR880260 (S-34); OR880263 (LZn4). The data is also provided within the related file which contain a previous publication by corresponding author.

## References

[1] Németh, M., Janda, T., Horváth, E., Páldi, E. & Szalai, G. Exogenous salicylic acid increases polyamine content but may decrease drought tolerance in maize. *Plant Sci.* **162**(4), 569–574 (2002).

[2] Yang, J., Kloepper, J. W. & Ryu, C. M. Rhizosphere bacteria help plants tolerate abiotic stress. *Trends Plant Sci.* **14**, 1–4 (2009). 19056309 10.1016/j.tplants.2008.10.004

[3] Glick, B. R. The enhancement of plant growth by free living bacteria. *Can. J. Microbiol.* **41**, 109–114 (1995).

[4] Bashan, Y. & Holguin, G. Proposal for the division of plant growth-promoting rhizobacteria into two classifications: Biocontrol-PGPB (plant growth-promoting bacteria) and PGPB. *Soil Biol. Biochem.* **30**, 1225–1228 (1998).

[5] Srivastava, S. et al. Effect of high temperature on *Pseudomonas putida* NBRI0987 biofilm formation and expression of stress sigma factor RpoS. *Curr Microbiol.* **56**, 453–457 (2008). 18219523 10.1007/s00284-008-9105-0

[6] Kloepper, J. W., Ryu, C. M. & Zhang, S. Induced systemic resistance and promotion of plant growth by *Bacillus* spp. *Phytopathology* **94**, 1259–1266 (2004). 18944464 10.1094/PHYTO.2004.94.11.1259

[7] Bensalim, S., Nowak, J. & Asiedu, S. K. A plant growth promoting rhizobacterium and temperature effects on performance of 18 clones of potato. *Am. J. Potato Res.* **75**, 145152 (1998).

[8] Sandhya, V., Ali, S. K. Z., Grover, M., Reddy, G. & Venkateswarlu, B. Effect of plant growth promoting *Pseudomonas* spp. on compatible solutes, antioxidant status and plant growth of maize under drought stress. *Plant Growth Regul.* **62**, 21–30 (2010).

[9] Fotovat, R., Valizadeh, M. & Toorchi, M. Association between water -use efficiency components and total chlorophyll content (SPAD) in wheat (*Triticum aestivum* L.) under well-watered and drought stressconditions. *J. Food Agric. Environ.* **5**(3&4), 225–227 (2007).

[10] Timmusk, S. & Wagner, E. G. H. The plant growth-promoting rhizobacterium *Paenibacillus polymyxa* induces changes in *Arabidopsis thalianan* gene expression: A possible connection between biotic and abiotic stress responses. *Mol. Plant-Microb. Interact.* **12**, 951–959 (1999). 10.1094/MPMI.1999.12.11.951 10550893

[11] Creus, C. M., Sueldo, R. J. & Barassi, C. A. Water relations and yield in Azospirillum-inoculated wheat exposed to drought in the field. *Can. J. Bot.* **82**, 273–281 (2004).

[12] Morrissey, J., Dow, J., Mark, L. & O’Gara, F. Are the microbes at the root solution to the world food production?. *EMBO Rep.* **5**, 922–926 (2004). 15459741 10.1038/sj.embor.7400263 PMC1299160

[13] Lugojan, S. & Ciulca, C. Evaluation of relative water content in whinter wheat. *J. Hortic. For. Biotechnol.* **15**, 173–177 (2011).

[14] Batool, T. et al. Plant growth promoting rhizobacteria alleviates drought stress in potato in response to suppressive oxidative stress and antioxidant enzymes activities. *Sci. Rep.* **10**, 16975 (2020). 33046721 10.1038/s41598-020-73489-z PMC7550571

[15] Khati. P., Mishra, P. K., Pal, R. & Kant, L. Isolation, screening and characterization of drought tolerant plant growth promoting bacteria from Indian Himalayas. *Pantnagar J. Res*. **22**(2) (2024).

[16] Shifa, S. et al. Research progress in the field of microbial mitigation of drought stress in plants. *Front. Plant Sci.* **13**, 870626 (2022). 35665140 10.3389/fpls.2022.870626 PMC9161204

[17] Teotia, P., Kumar, V., Kumar, M., Shrivastava, N. & Varma, A. Rhizosphere microbes: potassium solubilization and crop productivity–present and future aspects. In *Potassium Solubilizing Microorganisms for Sustainable Agriculture* (Springer, Berlin, 2016).

[18] Zhu, Q., Riley, W. J., Tang, J. & Koven, C. D. Multiple soil nutrient competition between plants, microbes, and mineral surfaces: Model development, parameterization, and example applications in several tropical forests. *Biogeosciences* **13**, 341–363 (2016).

[19] Kour, D. & Yadav, A. N. Microbe mediates mitigation of drought stress in crops. *Agric. Lett.* **1**, 79–82 (2020).

[20] Bilal, S. et al. Synergistic association of endophytic fungi enhances *Glycine max* L. resilience to combined abiotic stresses: Heavy metals, high temperature and drought stress. *Ind. Crops Prod.* **143**, 111931 (2020).

[21] Lopez, M. J. S., Dias-Filho, M. B. & Gurge, E. S. C. Successful plant growth-promoting microbes: Inoculation methods and abiotic factors. *Front Sustain. Food Syst.* **5**, 606454 (2021).

[22] Abbasi, S., Sadeghi, A. & Safaie, N. Streptomyces alleviate drought stress in tomato plants and modulate the expression of transcription factors ERF1 and WRKY70 genes. *Sci. Hortic.* **265**, 109206 (2020).

[23] Siebielec, S. et al. Impact of water stress on microbial community and activity in sandy and loamy soils. *Agronomy* **10**, 1429 (2020).

[24] Khan, N. & Bano, A. Exopolysaccharide producing rhizobacteria and their impact on growth and drought tolerance of wheat grown under rainfed conditions. *PLoS ONE* **14**(9), e0222302 (2019). 31513660 10.1371/journal.pone.0222302 PMC6742399

[25] Vurukonda, S. S., Vardharajula, S., Shrivastava, M. & Sk, Z. A. Enhancement of drought stress tolerance in crops by plant growth promoting rhizobacteria. *Microbiol. Res.* **184**, 13–24 (2016). 26856449 10.1016/j.micres.2015.12.003

[26] Sinsabaugh, R. L., Antibus, R. K. & Linkins, A. E. An enzymic approach to the analysis of microbial activity during plant litter decomposition. *Agric. Ecosyst. Environ.* **34**(1–4), 43–54 (1991).

[27] Kiss, S., Dragan-Bularda, M. & Radulescu, D. Soil polysaccharidases: activity and agricultural importance. In: *Soil Enzymes*, (Academic, London, 1978).

[28] Eivazi, F. & Tabatabai, M. A. Phosphatases in soils. *Soil Biol. Biochem.* **9**, 167–172 (1977).

[29] Dick, W. A., Cheng, L. & Wang, P. Soil acid and alkaline phosphatase activity as pH adjustment indicators. *Soil Biol. Biochem.* **32**(13), 1915–1919 (2000).

[30] Vinhal-Freitas, I. C., Wangen, D. R. B., Ferreira, A. S., Corrêa, G. F. & Wendling, B. Microbial and enzymatic activity in soil after organic composting. *Revista Brasileira de Ciência do Solo* **34**, 757–764 (2010).

[31] Khati, P. et al. Effect of nanozeolite and plant growth promoting rhizobacteria on maize. *3 Biotech* **8**(141), 1–12 (2018). 10.1007/s13205-018-1142-1 PMC5818361 29484280

[32] Khati, P., Chaudhary, P., Gangola, S., Bhatt, P. & Sharma, A. Nanochitosan induced growth of *Zea Mays* with soil health maintenance. *3 Biotech* **7**(81), 1–9 (2017). 10.1007/s13205-017-0668-y PMC5429309 28500403

[33] Khati, P. et al. Impact of agri-usable nanocompound on soil microbial activity: An indicator of soil health. *Clean Soil Air Water* **45**(5), 1–7 (2017).

[34] Agunbiade, V. F., Fadiji, A. E., Agbodjato, N. A. & Babalola, O. O. Isolation and characterization of plant-growth-promoting, drought-tolerant rhizobacteria for improved maize productivity. *Plants* **13**, 1298 (2024). 38794369 10.3390/plants13101298 PMC11125291

[35] Azeem, M., Haider, M. Z., Javed, S., Saleem, M. H. & Alatawi, A. Drought stress amelioration in maize (*Zea mays* L.) by inoculation of *Bacillus* spp. strains under sterile soil conditions. *Agriculture* **12**, 50 (2022).

[36] Ojuederie, O. B. & Babalola, O. O. Growth enhancement and extenuation of drought stress in maize inoculated with multifaceted ACC deaminase producing rhizobacteria. *Front. Sustain. Food Syst.* **6**, 1076844 (2023).

[37] Rashid, M. Assessment of Variation in Maize (*Zea mays* L.) for Response to Water Stress. *M.Sc. Thesis, Sher-e-Kashmir University of Agricultural Sciences and Technology* (2018).

[38] Junker, A. et al. Optimizing experimental procedures for quantitative evaluation of crop plant performance in high throughput phenotyping systems. *Front. Plant Sci.* **5**, 770 (2015). 25653655 10.3389/fpls.2014.00770 PMC4299434

[39] Karrou, M. & Maranville, J. W. Response of wheat cultivars to different soil nitrogen and moisture regime: II. Leaf water contents, stomatal conductance and photosynthesis. *J. Plant Nutr.* **8**, 777–791 (1995).

[40] Rao, M. V., Paliyath, G. & Ormrod, D. P. Ultraviolet-B- and ozoneinduced biochemical changes in antioxidant enzymes of *Avabidopsis thaliana*. *Plant Physiol.* **110**, 125–136 (1996). 8587977 10.1104/pp.110.1.125 PMC157701

[41] Nakano, Y. & Asada, K. Hydrogen peroxide is scavenged by ascorbate-specific peroxidase in spinach chloroplasts. *Plant Cell Physiol* **22**, 867–880 (1981).

[42] Jackson, M. L. *Soil Chemical Analysis* 1st edn. (Prentice Hall, 1973).

[43] Messer, W. & Johnson, C. H. Total viable counts. Pour plate technique. In *Encyclopedia of Food Microbiology*, Vol. 3, (Academic Press, London, 2000)

[44] Schnurer, J. & Rosswall, T. Fluorescein diacetate hydrolysis as a measure of total microbial activity in soil and litter. *Appl. Environ. Microbiol.* **6**, 1256–1261 (1982). 10.1128/aem.43.6.1256-1261.1982 PMC244223 16346026

[45] Eivazi, F. & Tabatabai, M. A. Glucosidases and galactosidases in soils. *Soil Biol. Biochem.* **20**, 601–606 (1988).

[46] Tabatabai, M. A. & Bremner, J. M. Use of p-nitrophenylphosphate for assay of soil phosphatase activity. *Soil Biol. Biochem.* **1**, 301–307 (1969).

[47] Tabatabai, M.A. 1994. Soil enzymes. In *Methods of Soil Analysis, Part 2: Microbiological and Biochemical Properties, SSSA Book Series No. 5* (Soil Science Society of America, Madison, 1994).

